# Chemistry of Advanced Nanomedicines in Cancer Cell Metabolism Regulation

**DOI:** 10.1002/advs.202001388

**Published:** 2020-08-07

**Authors:** Bowen Yang, Jianlin Shi

**Affiliations:** ^1^ State Key Laboratory of High Performance Ceramics and Superfine Microstructure Shanghai Institute of Ceramics Chinese Academy of Sciences Shanghai 200050 P. R. China; ^2^ Center of Materials Science and Optoelectronics Engineering University of Chinese Academy of Sciences Beijing 100049 P. R. China

**Keywords:** cancer, material chemistry, metabolism regulation, nanomedicine, synergistic therapy

## Abstract

Tumors reprogram their metabolic pathways to meet the bioenergetic and biosynthetic demands of cancer cells. These reprogrammed activities are now recognized as the hallmarks of cancer, which not only provide cancer cells with unrestricted proliferative and metastatic potentials, but also strengthen their resistance against stress conditions and therapeutic challenges. Although recent progress in nanomedicine has largely promoted the developments of various therapeutic modalities, such as photodynamic therapy, photothermal therapy, nanocatalytic therapy, tumor‐starving/suffocating therapy, etc., the therapeutic efficacies of nanomedicines are still not high enough to achieve satisfactory tumor‐suppressing effects. Therefore, researchers are obliged to look back to the essence of cancer cell biology, such as metabolism, for tailoring a proper therapeutic regimen. In this work, the characteristic metabolic pathways of cancer cells, such as aerobic respiration, glycolysis, autophagy, glutaminolysis, etc. are reviewed, to summarize the very recent advances in the smart design of nanomedicines that can regulate tumor metabolism for enhancing conventional therapeutic modalities. The underlying chemistry of these nanomedicines by which tumor metabolism is harnessed, is also discussed in a comprehensive manner. It is expected that by harnessing tumor metabolism cancer nanotherapeutics will be substantially improved in the future.

## Graphical abstract

Cancer cell metabolism regulation has been achieved by advanced nanomedicines for enhancing various cancer therapeutic modalities. This work summarizes recent advances in the fabrication of metabolism‐regulating nanomedicines and the associated chemical strategies through which the core of tumor biology would be turned into cancer's Achilles heel.

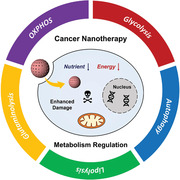

## 1 Introduction

Schrödinger's book *What is Life? The Physical Aspect of the Living Cell*, which was first published in 1944, has created a wave of enthusiasm among scientists to explore how human beings can take advantage of the fundamentals of cell biology to solve the problems encountered in life.^[^ ^1^ ^]^ For example, the improvement of clinical therapies requires us to have a better understanding on cell biology for elevating therapeutic outcomes in organismal levels by regulating specific cellular behaviors. As the central science of cell biology, cell metabolism discloses the mechanism of chemical reactions taking place during various cellular bioenergetic and biosynthetic processes, which also inspires chemists to design rational chemical strategies for metabolism regulation.

Since the first demonstration of aerobic glycolysis of cancer cells by Warburg in 1924,^[^ ^2^ ^]^ great efforts have been devoted to investigating the characteristics of cancer cell metabolism for designing possible therapeutic strategies. It has now become clear that the “Warburg effect” presents only a tip of the iceberg regarding the metabolic rearrangements of cancer cells, which involve not only aerobic glycolysis but also a high glutamine consumption, a limited autophagy level, elevated rates of lipid biosynthesis, etc.^[^ ^3^ ^]^ The varied metabolic activities of cancer cells support their anabolism for growth during nutrient repletion, catabolism for survival during nutrient limitation, and fortification of redox homeostatic systems for detoxication under oxidative stress.^[^ ^4^ ^]^ These reprogrammed activities not only provide cancer cells with significant proliferative and metastatic potentials, but also strengthen their resistance against various stress conditions and therapeutic challenges.^[^ ^5^ ^]^ Therefore, chemistries targeting these biological processes by regulating the cellular activities or concentrations of enzymes, substrates and metabolites, may open diverse therapeutic windows.^[^ ^6^ ^]^

Recent nanotechnology in biomedicine has promoted the development of numbers of nanosystems for advancing various cancer therapeutic modalities, such as chemotherapy, radiation therapy, gene therapy, photodynamic therapy (PDT), etc.^[^ ^7^ ^]^ Although these nanosystems have been demonstrated with improved bioavailability compared to conventional molecular drugs, however, current nanomedicines still suffer from unsatisfactory therapeutic efficacy resulting from the relatively high chemoresistance of cancer cells enabled by their reprogrammed metabolism. These aggressive while tough cancer cells increase their uptake of building blocks (glucose and amino acids) as well as oxygen from the tumor microenvironment, to maintain the balance between macromolecular synthesis and high adenosine triphosphate (ATP) level for cell survival under harsh conditions triggered by extrinsic therapeutic interventions such as anticancer drugs, genes, reactive oxygen species (ROS), and heat,^[^ ^6^ ^]^ finally compromising the therapeutic effects of nanomedicines. This situation leads us to reconsider therapeutic strategies in addition to the conventional design rationale for intensified tumor‐specific toxicity of nanomedicine. We should not only pay attention to the construction of nanosystems with high physical/chemical performances, but also return to and focus on the basic laws of biological processes in cancer cells to solve the most essential issues by rational metabolic regulation.

**Figure 1:**
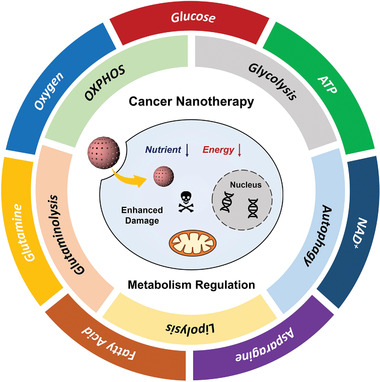
Schematic illustrations of metabolism regulation strategies for augmenting cancer therapeutics. The core shows the cell metabolism regulation by engineered nanomedicine; the inner circle demonstrates several main metabolic pathways of cancer cells that can be regulated to reduce their resistances to therapeutic interventions, while the outer circle indicates several main building materials or metabolites during these bioenergetic or biosynthetic processes. OXPHOS, oxidative phosphorylation; ATP, adenosine 5’‐triphosphate; NAD^+^, oxidized form of nicotinamide adenine dinucleotide.

In the past several years, significant advances have been made in the design of nanomedicines with cancer cell metabolism‐regulating capabilities. This field grows fast, accompanied by accumulating experimental results evidencing improved cancer therapeutic outcomes. However, our current understanding on the underlying material chemistry of these nanomedicines, the chemical essence of metabolic process in cancer cells, as well as the synergetic effect between them for favored tumor‐specific therapeutic efficacies, remains preliminary and unclear. Therefore, in this review, we will concentrate on the metabolic pathways of cancer cells, such as aerobic respiration, glycolysis, autophagy, glutaminolysis, etc. (**Figure** **1**), to provide a comprehensive elucidation on the nanomedicine‐based metabolism regulation strategies for augmenting cancer therapeutics. The detailed biochemical mechanism for each metabolic pathway will be clarified in each corresponding section, followed by in‐depth discussions on the current achievements and future possible developments in regulating these metabolic pathways by nanomedicines. We hope that by focusing on these strategies the core of tumor biology might be turned into cancer's Achilles heel.

## 2 Aerobic Respiration

Aerobic respiration is a metabolic process in which cells, with the participation of oxygen, completely oxidize, and decompose several organic matters (carbohydrates, fats, and then proteins), release carbon dioxide and water as well as a large amount of energy at the same time. The mitochondrial oxidative phosphorylation (OXPHOS) is the most important step of aerobic respiration, during which nicotinamide adenine dinucleotide (NADH) and reduced flavine adenine dinucleotide (FADH_2_) generated in the previous two steps (glycolysis and tricarboxylic acid cycle) are consumed in the presence of oxygen for generating ATP (28 ATP per metabolized glucose). Herein, oxygen is an essential nutrient and a key substrate for mitochondrial respiration, while reduced oxygen availability will compromise the efficiency of energy production.

**Figure 2:**
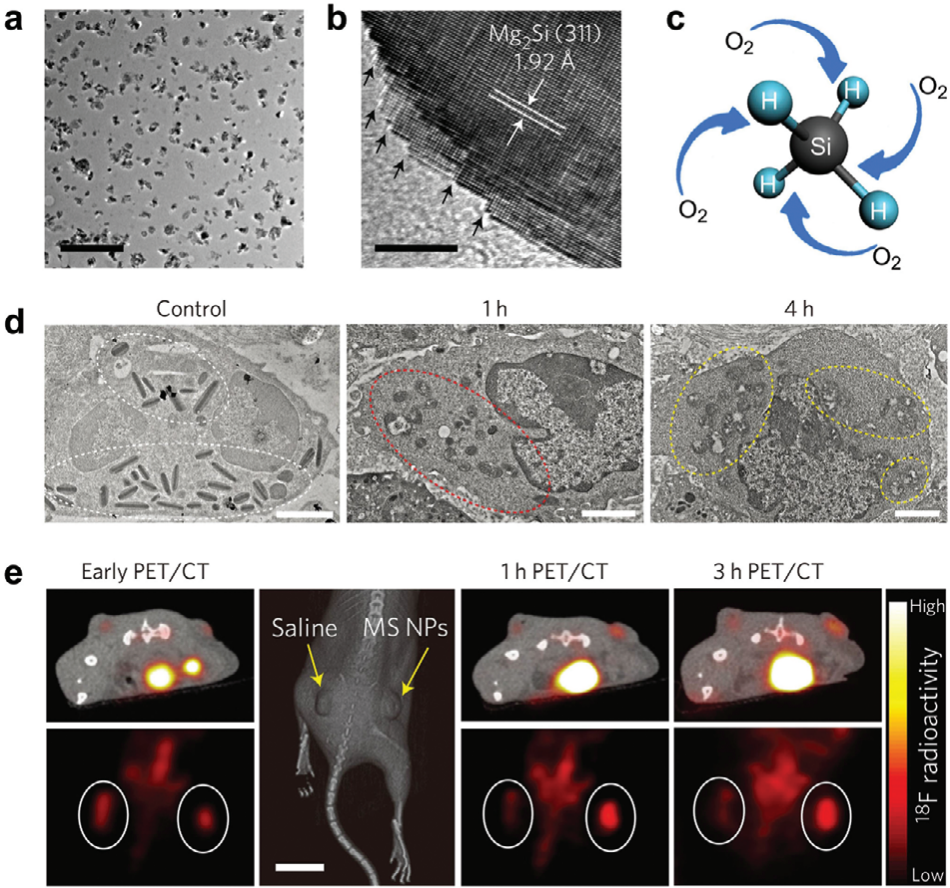
Magnesium silicide nanoparticles as a deoxygenation agent for inhibiting mitochondrial respiration of cancer cells. a) Transmission electron microscopy (TEM) image of highly dispersed Mg_2_Si nanoparticles. Scale bar, 500 nm. b) High‐resolution TEM image showing the serrated edge of a single Mg_2_Si nanoparticle. Scale bar, 5 nm. c) Mechanism of deoxygenation reaction by Mg_2_Si nanoparticles. d) Bio‐TEM images of MCF‐7 cells treated with Mg_2_Si nanoparticles for prolonged durations, which show morphological damage of suffocated mitochondria. e) ^18^F‐labeled fluoromisonidazole positron emission tomography (PET)/computed tomography (CT) images before and after different‐treatments showing a significantly enhanced hypoxia after Mg_2_Si nanoparticle (denoted MS NP in this figure) injection. Reproduced with permission.^[^ ^10^ ^]^ Copyright 2017, the Authors, published by Springer Nature.

To meet the increasing bioenergetic demands for cell growth and proliferation, tumors consume more oxygen than normal tissue and thus develop oxygen‐deprived microenvironments.^[^ ^8^ ^]^ However, under the hypoxic conditions cancer cells tend to use oxygen‐sensing pathways to adapt to microenvironmental stresses for sustaining metabolism.^[^ ^9^ ^]^ This hallmark of cancer inspires scientists to further deplete intratumoral oxygen for inhibiting mitochondrial respiration of cancer cells. Our research group has reported polyvinyl pyrrolidone (PVP)‐modified Mg_2_Si nanoparticles as a deoxygenating agent to realize tumor‐suffocating therapy (**Figure** **2**).^[^ ^10^ ^]^ In the mildly acidic tumor microenvironment, the Mg_2_Si nanoparticles can be activated specifically to scavenge environmental oxygen via the following chemical reactions:

(1) $$\begin{array}{c} Mg_{2}\mathrm{Si}+4H^{+}\to 2Mg^{2+}+\mathrm{Si}H_{4} \end{array}$$

(2) $$\begin{array}{c} \mathrm{Si}H_{4}+2O_{2}\to 2H_{2}O+\mathrm{Si}O_{2} \end{array}$$

In all:

(3) $$\begin{array}{c} Mg_{2}\mathrm{Si}+4H^{+}+2O_{2}\to 2Mg^{2+}+2H_{2}O+\mathrm{Si}O_{2} \end{array}$$

The intermediate silane (SiH_4_) is nontoxic but highly reactive with O_2_ to promote intratumoral deoxygenation. Moreover, the reaction product SiO_2_ aggregates could block tumor capillaries and exacerbate tumor hypoxia by cutting off blood supplies. The mitochondria in cancer cells after Mg_2_Si nanoparticle treatment present serious swelling, membrane breakage, and plasma spillover, which results from the inhibition on their respiratory functions after local deoxygenation, finally leading to cellular necrosis and/or apoptosis. In vivo imaging by ^18^F‐labeled fluoromisonidazole positron emission tomography (PET)/computed tomography (CT) further evidenced high oxygen‐scavenging performance of Mg_2_Si nanoparticle, indicating its future translation potential. Inspired by our work, Chen et al. have also integrated Mg_2_Si nanoparticles with hypoxia‐responsive drug tirapazamin (TPZ) for concurrent tumor‐suffocation and chemotherapy.^[^ ^11^ ^]^

**Figure 3:**
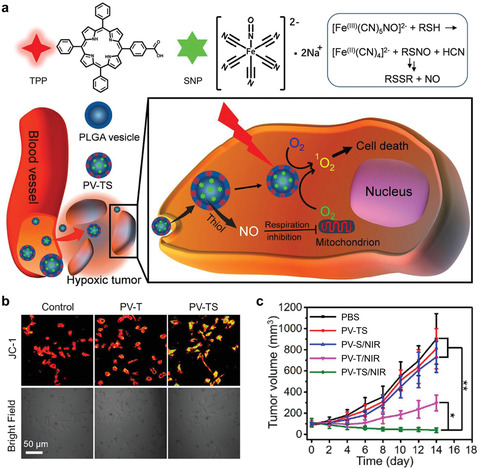
NO‐generating nanoparticles to inhibit aerobic respiration and elevate cellular O_2_ level for enhancing PDT. a) Schematic illustration for the composition and cellular working mechanism of NO‐generating nanoparticles. TPP, tetraphenylporphyrin; SNP, sodium nitroprusside; RSH, thiol substrates (such as glutathione and cysteine); RSNO, S‐nitrosothiol; PV‐TS, SNP and TPP coloaded polymeric nanovesicles. b) Semiquantitative determination of mitochondrial transmembrane potential ΔΨ_m_ by fluorescence 5,5’,6,6’‐tetrachloro‐1,1’,3,3’‐tetraethylbenzimidazolocarbocyanine iodide (JC‐1) analysis. The J‐aggregate (red fluorescence) will form from JC‐1 monomer (green fluorescence) when ΔΨ_m_ increases, and this process is reversible. PV‐T, TPP‐loaded polymeric nanovesicles. c) Relative tumor volume of mice after different treatments. *P < 0.05, **P < 0.01. Reproduced with permission.^[^ ^13^ ^]^ Copyright 2019, American Chemical Society.

Nitric oxide (NO) is a prominent gas transmitter in biosystem, which can restrain cell respiration by inhibiting complex IV in mitochondrial respiratory chain in competition with oxygen.^[^ ^12^ ^]^ This unique chemical property of NO has been utilized as an alternative strategy for enabling tumor‐suffocating therapy, by delivering NO‐generating nanosystems into cancer cells. As a typical paradigm, Yu et al. have constructed a polymeric nanosystem coloaded with a hydrophilic NO donor sodium nitroprusside (SNP) and a hydrophobic photosensitizer tetraphenylporphyrin (TPP) for tumor suffocation‐enhanced PDT (**Figure** **3**).^[^ ^13^ ^]^ SNP can react with the thiol compounds overexpressed in cancer cells to form S‐nitrosothiol, promoting the NO production to enable efficient mitochondrial respiration inhibition, during which the oxygen consumption is reduced and the tumor hypoxia is mitigated, finally favoring PDT processes triggered by photosensitization of TPP. Cellular experiments evidenced a decreased mitochondrial membrane potential (ΔΨ_m_), a reduced ATP level and an elevated singlet oxygen (^1^O_2_) production after the treatment with the composite nanosystem, accompanied by a significant reduction of cell viability, indicating that such a NO‐generating nanosystem is efficient to block aerobic respiration pathway and enhance cancer treatment. Following this work, Wei et al. also fabricated a near‐infrared (NIR) light activated nanosystem by loading polydopamine nanoparticles with a NO donor (N,N’‐di‐sec‐butyl‐N,N’‐dinitroso‐1,4‐phenylenediamine, BNN6) and an anticancer drug doxorubicin (DOX) for tumor‐suffocating/chemo/photothermal triple‐combination therapy.^[^ ^14^ ^]^

Lactate is a characteristic metabolite of glucose released after glycolysis, which has been recently demonstrated to be capable of entering another cell to fuel OXPHOS process, a phenomenon known as “Lactate shuttle” or “reversed Warburg effect.”^[^ ^5^ ^]^ Therefore, in addition to the previous strategies of deoxygenation or NO generation, reducing extrinsic lactate availability to interfere the lactate‐fueled aerobic respiration of cancer cells would also be a promising therapeutic strategy. Zhang et al. have engineered porous Zr (IV)‐based porphyrinic metal‐organic framework (MOF) nanoparticles with *α*‐cyano‐4‐hydroxycinnamate, which is able to reduce the extrinsic lactate uptake by inhibiting the expression of monocarboxylate transporter 1 (MCT1) on cancer cells, thus restraining lactate‐fueled aerobic respiration.^[^ ^15^ ^]^ Moreover, this strategy is also capable of enhancing PDT due to the reduced oxygen consumption and mitigated tumor hypoxia after respiration inhibition. This work reveals the importance of reducing extrinsic lactate availability and may promote the emergence of other lactate‐scavenging nanosystems for cancer therapy.

**Figure 4:**
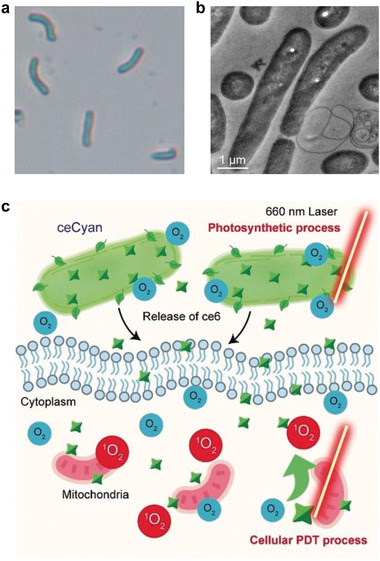
Photosensitizer‐containing cyanobacteria for photosynthetic tumor oxygenation and consequently enhanced PDT. a) Optical and b) TEM image of cyanobacteria. c) Schematic illustration of the cellular oxygenation using engineered cyanobacteria (ceCyan) favoring aerobic respiration and PDT. Reproduced with permission.^[^ ^17^ ^]^ Copyright 2020, Wiley‐VCH.

The previous strategies focused on the blockage of aerobic respiration pathway, either by directly triggering tumor‐suffocating therapeutic effect, or by reducing the oxygen consumption for facilitating other therapeutic modalities. However, from the viewpoint of intracellular oxygen level, an approach of directly elevating oxygen availability to boost tumor therapy such as PDT that requires oxygen as reactant, can also be easily conceived. Such an approach seems contradictory to the previously demonstrated deoxygenation strategy, but may also work in a different mechanism: increasing oxygen availability is a supplementary means to augment other oxygen‐dependent therapeutic modalities, while in comparison, reducing intratumoral oxygen availability can directly suffocate tumors. Given the basis of several mechanisms of tumor resistance to therapeutic interventions is in the aberrant aerobic respiration of cancer cells, the reactivation of a more “normal” metabolic activity could revert tumors to being sensitive to therapies.^[^ ^16^ ^]^ We have recently designed a photosensitizer‐containing cyanobacteria to promote photosynthetic tumor oxygenation for enhancing PDT (**Figure** **4**).^[^ ^17^ ^]^ By feeding tumor with a large amount of oxygen by the photosynthetic oxygen production of cyanobacteria, the tumor metabolism was normalized and the hypoxia was mitigated, finally elevating the PDT efficacy to a large extent. Zheng et al. have also prepared a photosynthetic leaf‐inspired abiotic/biotic nanothylakoid system for efficient O_2_ generation in tumor.^[^ ^18^ ^]^ Under 660 nm laser irradiation, it was found that the composite nanosystem could not only normalize the entire metabolic network of cancer cells (especially tricarboxylic acid cycle), but also amend the abnormal structure and function of tumor vasculature. Improved therapeutic outcomes were observed after treatment with the nanomedicine in conjunction with PDT or antiangiogenesis therapy, demonstrating that increasing intratumoral oxygen availability to normalize tumor metabolism is also a promising auxiliary means for enhancing cancer therapy.

Based on these discussions, it should be noted here that a proper respiration regulation strategy could be designed based on specific situations. To obtain direct therapeutic effect, using deoxygenating agent to deprive intratumoral oxygen is more appropriate. Alternatively, however, reducing oxygen consumption or increasing oxygen supply could be equally effective in the synergetic cancer therapy in combination with other oxygen‐dependent modalities.

## 3 Glycolysis

Glycolysis is a kind of metabolic process that converts glucose to pyruvate, generating two moles of pyruvate, two moles of NADH and two moles of ATP per mole of glucose. Cancer cells are characterized by increased glycolysis compared with normal ones regardless of cellular oxygen concentration, after which the pyruvate is reduced to lactate by lactate dehydrogenase (LDH) subsequently.^[^ ^16^ ^]^ It is noted that although glycolysis is a less efficient metabolic pathway for energy generation compared with OXPHOS (28 ATP per glucose), however, the rate of ATP generation from the glycolytic pathway is approximately 100‐fold faster than that from OXPHOS.^[^ ^19^ ^]^ Therefore, to meet the increasing bioenergetic demands, cancer cells consume over 10 times more glucose than normal ones and increase the rate of glycolytic reactions by over 30 folds.^[^ ^19^ ^]^

**Figure 5:**
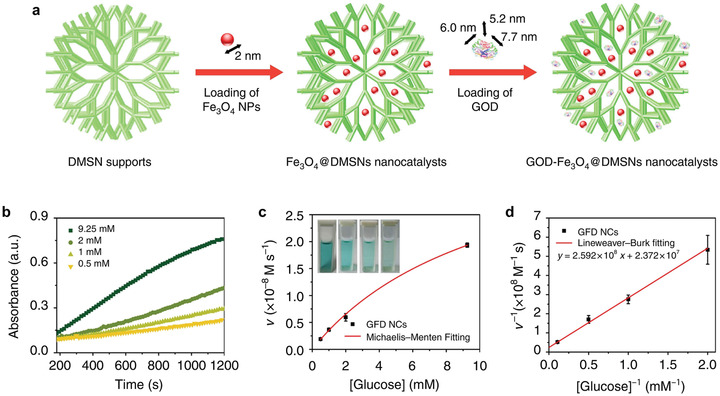
Glucose oxidase‐loaded nanosystem to deplete cellular glucose for starvation‐augmented nanocatalytic cancer therapy. a) Synthetic procedure of Fe_3_O_4_ and glucose oxidase (GOD) coloaded dendritic mesoporous silica nanoparticles (DMSNs). b) Time‐course absorbance of GOD‐Fe_3_O_4_@DMSNs nanocatalysts (denoted GFD NCs) upon the addition of varied concentrations of glucose. c) Michaelis–Menten kinetics of GFD NCs based on (b). d) Lineweaver–Burk plotting of GFD NCs based on (c). Reproduced with permission.^[^ ^20^ ^]^ Copyright 2017, Springer Nature.

Based on such metabolic alterations of cancer cells, till now, two typical strategies have been conceived to inhibit glycolysis for triggering tumor‐therapeutic effect. The first one is to deplete intratumoral glucose by using specific catalysts to remove the initial reactant of glycolytic reactions. We have designed a mesoporous silica nanoparticle (MSN)‐based nanosystem coloaded with glucose oxidase and ultrasmall Fe_3_O_4_ nanoparticles for starvation‐augmented nanocatalytic cancer therapy (**Figure** **5**).^[^ ^20^ ^]^ The glucose oxidase as a natural enzyme can catalyze cellular glucose into hydrogen peroxide (H_2_O_2_), which not only leads to severe tumor starvation, but also favor subsequent Fenton‐like reactions triggered by Fe_3_O_4_ nanoparticles, during which the H_2_O_2_ generated in the former reaction were converted into highly toxic hydroxyl radicals (•OH),^[^ ^21^ ^]^ further enhancing antitumor effect in a synergetic way. Such sequential reactions can be illustrated by the following equations:

(4) $$\begin{array}{c} \mathrm{Glucose}+O_{2}+H_{2}O\overset{\mathrm{glucose}\;\mathrm{oxidase}}{\to }\mathrm{Gluconic}\;\mathrm{acid}+H_{2}O_{2} \end{array}$$

(5) $$\begin{array}{c} H_{2}O_{2}\overset{Fe_{3}O_{4}}{\to }•\mathrm{OH}+•O_{2}H \end{array}$$

Following this work, we have also used Au nanoparticles as a glucose oxidase to mimic the natural enzyme for a more stable catalytic activity and an elevated glucose‐depleting efficiency.^[^ ^22^ ^]^ Such a strategy by catalytic glucose scavenging has also been combined with other therapeutic modalities. For example, Fan et al. have engineered glucose oxidase and the NO donor arginine in the hollow mesoporous organosilica nanoparticle (HMONs) for synergistic tumor‐starving/gas therapy.^[^ ^23^ ^]^ The arginine can react with the gluconic acid and H_2_O_2_ generated during the catalytic reaction triggered by glucose oxidase, promoting the generation of NO (Equation 6), which have been demonstrated in the previous section that can inhibit cell respiration.

(6) $$\begin{array}{c} \mathrm{Arginine}+H_{2}O_{2}\overset{H^{+}}{\to }\mathrm{Citrulline}+\mathrm{NO}+H_{2}O \end{array}$$

**Figure 6:**
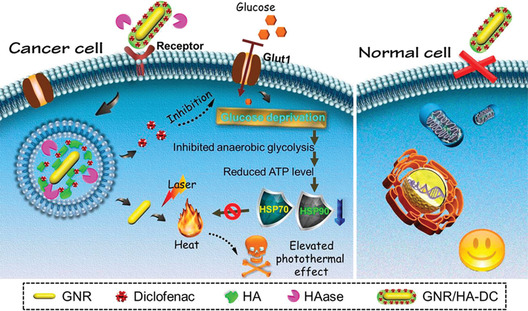
GLUT1 inhibitor‐loaded gold nanorods to favor glucose deprivation and inhibit heat shock protein expression for enhancing PTT. HSP, heat shock protein; GNR, gold nanorod; HA, hyaluronic acid; HAase, hyaluronidase; DC, diclofenac. Reproduced with permission.^[^ ^24^ ^]^ Copyright 2017, American Chemical Society.

**Figure 7:**
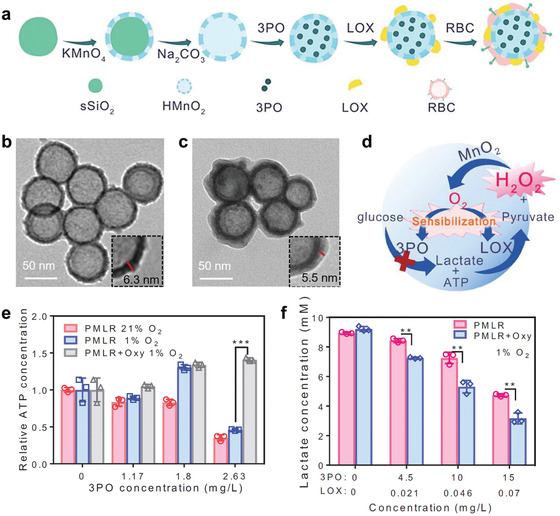
Glycolysis inhibition in combination with lactic acid exhaustion to modulate acidic microenvironment for enhancing immunotherapy. a) Synthetic procedure of the composite nanomedicine (denoted PMLR). 3PO, 3‐(3‐pyridinyl)‐1‐(4‐pyridinyl)‐2‐propen‐1‐one; LOX, lactate oxidase; RBC, red blood cell; HMnO_2_, hollow MnO_2_. TEM images of b) HMnO_2_ nanoparticles and c) PMLR nanoparticles. d) Schematic illustration of the closed loop of PMLR nanosystem for glycolysis inhibition and lactate consumption. e) Relative ATP concentration of B16F10 cells after treatment with PMLR nanoparticles under normoxia, hypoxia or hypoxia with the addition of H_2_O_2_ scavenger oxyresveratrol (Oxy). ***P < 0.001. f) Lactate concentration of B16F10 cells after treatment with PMLR nanoparticles under hypoxia with or without the addition of Oxy. **P < 0.01. Reproduced with permission.^[^ ^26^ ^]^ Copyright 2019, Wiley‐VCH.

The second approach is to use specific glycolysis inhibitors to block glycolytic reaction pathways. Zhang et al. have decorated GLUT1 inhibitor diclofenac (DC) on gold nanorods to result in reduced glucose uptake, blocked glycolysis, decreased ATP level and hampered heat shock protein (HSP) expression, finally the potency of gold nanorod‐based photothermal therapy (PTT) has been significantly elevated (**Figure** **6**).^[^ ^24^ ^]^ Inspired by this work, Yin et al. developed a nanosystem loaded with a siRNA against pyruvate kinase M2 (siPKM2) and the photothermal agent indocyanine green (ICG), by which tumor glycolysis and the subsequent ATP supply were inhibited, thus overcoming the thermo‐resistance of cancer cells to augment ICG‐mediated photothermal tumor ablation.^[^ ^25^ ^]^ Gao et al. have also fabricated a red blood cell membrane‐coated hollow MnO_2_‐based nanosystem embedded with lactate oxidase (LOX) and a glycolysis inhibitor 3‐(3‐pyridinyl)‐1‐(4‐pyridinyl)‐2‐propen‐1‐one (3PO) for intra/extracellular lactic acid exhaustion as well as glycolysis inhibition, which can further normalize tumoral acidic microenvironment for enhancing immunotherapy (**Figure** **7**).^[^ ^26^ ^]^ Interestingly, the components in the composite nanosystem synergize with each other in enhancing therapeutic effect collectively, as illustrated in the following equations:

(7) $$\begin{array}{c} \mathrm{Mn}O_{2}+H_{2}O_{2}+H^{+}\to Mn^{2+}+O_{2}+H_{2}O \end{array}$$

(8) $$\begin{array}{c} \mathrm{Glucose}\to \mathrm{Lactate}\;\left(\mathrm{Inhibited}\;\mathrm{by}\;3\mathrm{PO}\right) \end{array}$$

(9) $$\begin{array}{c} \mathrm{Lactate}+O_{2}\overset{\mathrm{LOX}}{\to }\mathrm{Pyruvic}\;\mathrm{acid}+H_{2}O_{2} \end{array}$$

The hollow MnO_2_ carrier can react with H_2_O_2_ overexpressed in cancer cells to favor the generation of O_2_, which sensitizes 3PO and LOX to inhibit the phosphofructokinase of glycolysis and promote lactate scavenging, respectively. Moreover, H_2_O_2_ can be generated during the oxidation of lactate, which further promotes O_2_ generation by reacting with MnO_2_. The synergistic operation among oxygen generation, glycolysis inhibition and lactate scavenging is evidenced to significantly elevate therapeutic outcome both in vitro and in vivo, demonstrating that this strategy can be further developed for enhancing cancer therapeutic modalities. Based on the discussions on the “Lactate shuttle” effect in the previous section in which lactate can enter another cell to fuel OXPHOS process, this therapy is expected to inhibit glycolysis and OXPHOS concurrently, but more evidences should be further provided.

**Figure 8:**
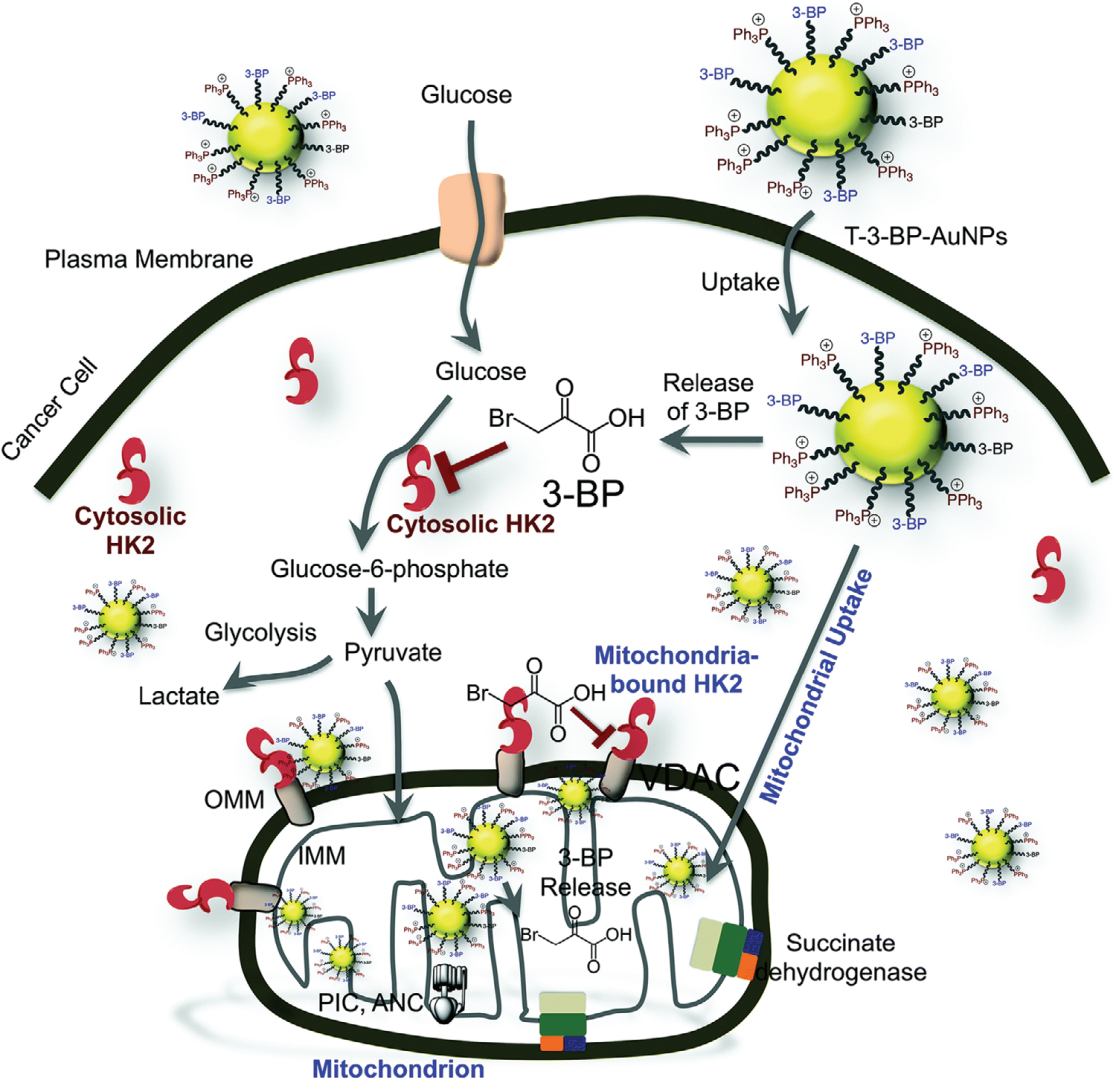
Mitochondria‐targeted delivery of 3‐bromopyruvate (3‐BP) for concurrent inhibition of glycolysis and OXPHOS. VDAC, voltage‐dependent anion channel; IMM, inner mitochondrial membrane; OMM, outer mitochondrial membrane; PIC, phosphate carrier; ANC, adenine nucleotide carrier. Reproduced with permission.^[^ ^28^ ^]^ Copyright 2015, Royal Society of Chemistry.

Cancer cells have been demonstrated to be capable of switching their metabolic pathways between glycolysis and OXPHOS to adapt to endogenous and exogenous metabolic challenges.^[^ ^27^ ^]^ Therefore, concurrent inhibitions of the two pathways by nanomedicines may achieve synergistic effect to significantly promote energy deprivation of tumor compared with single metabolic inhibition. This combined strategy was demonstrated to be effective in suppressing tumor growth by Dhar et al. in 2015, who fabricated mitochondria‐targeted gold nanoparticles decorated with 3‐bromopyruvate (3‐BP) and delocalized lipophilic triphenylphosphonium cations for efficient energy blockage (**Figure** **8**).^[^ ^28^ ^]^ 3‐BP is a brominated derivative of pyruvic acid and an alkylating agent that selectively inhibits hexokinase 2 (HK2) of glycolysis. Moreover, 3‐BP can also suppress the mitochondrial succinate dehydrogenase, mitochondrial phosphate carrier and adenine nucleotide carrier, finally inhibiting OXPHOS. Cellular experiments also indicated significantly reduced lactate and ATP productions, and such a therapeutic effect can be further amplified by the photothermal effect of gold nanoparticles. Wang et al. have also achieved codelivery of albendazole and nanosilver for efficient energy deprivation of MCF‐7 tumor xenograft, in which albendazole is a typical antiglycolytic agent while nanosilver is efficient to inhibit mitochondrial respiration.^[^ ^29^ ^]^ A recent follow‐up study by these scientists has also achieved brain delivery of the two agents for glioma treatment.^[^ ^30^ ^]^

**Figure 9:**
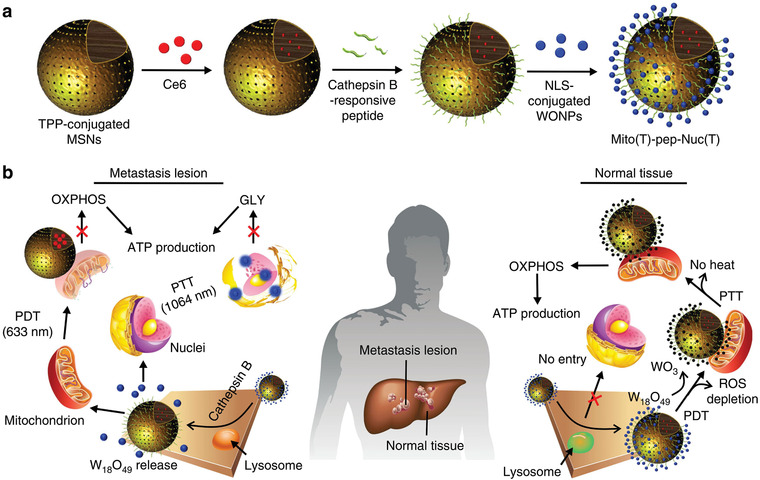
Tumor‐specific therapy by concurrently inhibiting glycolysis and OXPHOS in cancer cells. a) Synthetic procedure of the mitochondria and nucleus dual‐targeting nanosystem. NLS, nuclear localization sequence; WONP, W_18_O_49_ nanoparticle. b) Schematic illustration showing the working mechanism of the composite nanomedicine. GLY, glycolysis. Reproduced with permission.^[^ ^31^ ^]^ Copyright 2019, Springer Nature.

However, as normal cells also rely on OXPHOS for ATP production, this combined approach may inevitably lead to side effect to normal tissues. To overcome this limitation, Gu et al. has designed a mitochondria and nucleus dual‐targeting nanosystem to enable tumor‐specific energy depletion for eradication of liver metastasis (**Figure** **9**).^[^ ^31^ ^]^ This nanosystem was fabricated by conjugating nucleus‐targeting W_18_O_49_ nanoparticles on mitochondria‐selective photosensitizer (Ce6)‐loaded MSNs through a cathepsin B‐responsive peptide, which can be cleaved specifically in cancer cells, leading to the separation of the two types of nanoparticles. W_18_O_49_ nanoparticle with photothermal conversion capability enables the PTT of nuclei, interfering the expression of LDHA and restraining glycolysis, while Ce6‐loaded MSN enables PDT of mitochondria, thus inhibiting OXPHOS. Comparatively, in normal cells such as hepatocytes, the photodynamically generated ^1^O_2_ by Ce6‐loaded MSNs can be stoichiometrically consumed by W_18_O_49_ nanoparticles, which concurrently abolishes the functions of PTT and PDT, leaving the glycolysis and OXPHOS unaffected. This study provides a paradigm for metabolism‐inhibiting therapy with enhanced tumor specificity.

## 4 Autophagy

Autophagy is an evolutionarily conserved catabolic mechanism by which cells capture cytoplasmic components and deliver them to lysosomes for degradation.^[^ ^32^ ^]^ It involves the formation of specialized double‐membraned vesicles known as autophagosomes, which can progressively sequester autophagic cargos such as proteins, lipids and organelles and deliver them to lysosomes by one‐to‐one membrane fusion, forming autolysosomes. The inner autophagic cargos will be degraded by lysosomal hydrolases to generate nutrients such as amino acids, acetyl‐CoA and pyruvate, and these building blocks are recycled to replenish tricarboxylic acid cycle intermediates through anaplerotic reactions for supporting ATP generation and/or new biomacromolecule synthesis. It is noted that autophagy also serves as a self‐protection mechanism of cells against extrinsic adverse conditions such as nutrient deprivation, hyperthermia, oxidative stress, etc., by generating compensatory nutrients or degrading damaged or dangerous cellular components for maintaining homeostasis.^[^ ^33^ ^]^

**Figure 10:**
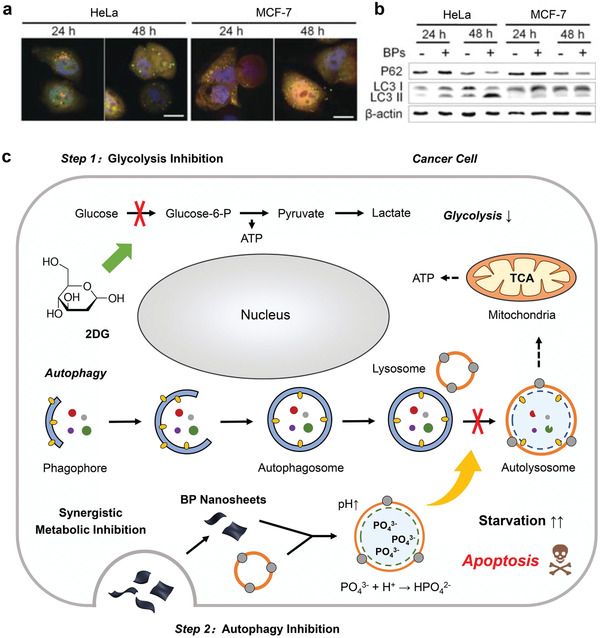
BP nanosheets with autophagy‐inhibiting capabilities for cancer therapeutic applications. a) Confocal fluorescence images of HeLa and MCF‐7 cells expressing mRFP‐GFP‐LC3 after treatment with BP nanosheets for 24 and 48 h. Aberrant autophagosome accumulation (yellow) can be visualized, which attribute to the lysosomal blockage by BP nanosheets. Scale bar, 10 µm. b) Immunoblot analyses for the expressions of p62 and LC3 in HeLa and MCF‐7 cells after treatments with BP nanosheets for 24 and 48 h. *β*‐actin was used as a loading control. Reproduced with permission.^[^ ^36^ ^]^ Copyright 2019, Wiley‐VCH. c) Schematic illustration for the cellular mechanism of the synergy between 2DG and BP nanosheets that enables concurrent glycolysis inhibition and autophagy blockage for exacerbating cancer cell starvation. Reproduced with permission.^[^ ^39^ ^]^ Copyright 2020, Wiley‐VCH.

For cancer therapy, such a metabolic pathway endows cancer cells with resistances against various therapeutic interventions.^[^ ^32^ ^]^ For example, under starvation therapy, cancer cells will activate autophagy to “eat” and “digest” their dispensable components to compensate for the absence of nutrient and energy for survival, compromising the therapeutic efficacy.^[^ ^33^ ^]^ Therefore, inhibition of autophagy is a promising strategy to augment current cancer therapeutic modalities. Recent advances in nanotechnology have promoted the exploration of various nanoparticles with autophagy‐regulating capabilities, and several of them have been demonstrated to possess autophagy‐inhibiting function owing to their unique chemical properties.^[^ ^34^ ^]^ As a typical paradigm, a recent study has indicated that black phosphorus (BP) nanosheets, which have been extensively used in various biomedical applications,^[^ ^35^ ^]^ can block the lysosomal degradation of cancer cells specifically and lead to aberrant autophagosome accumulation (**Figure** **10**a,b).^[^ ^36^ ^]^ As cancer cells are characterized with high ROS levels,^[^ ^37^ ^]^ BP nanosheets with single phosphorus component are easily oxidized and degraded in cancer cells, promoting the elevation of cellular phosphate anions (PO_4_^3−^) level. PO_4_^3−^ is alkaline in aqueous solution according to the Brønsted‐Lowry acid‐base theory,^[^ ^38^ ^]^ which can deplete lysosomal H^+^ by generating conjugated acid anions (HPO_4_^2−^), compromising the catalytic function of lysosomal hydrolases and the degradation efficiency of autophagosomes.

Therefore, BP nanosheets is expected to enhance cancer therapeutic modalities by blocking autophagy. We have recently reported a combinational therapeutic strategy by concurrent uses of BP nanosheets and 2‐deoxy‐d‐glucose (2DG), enabling enhanced tumor starvation (Figure 10c).^[^ ^39^ ^]^ 2DG is a typical antiglycolytic agent that can inhibit HK of glycolysis for triggering tumor starvation,^[^ ^40^ ^]^ while the downstream autophagic flux and compensatory energy supply are blocked by BP nanosheets. In this way, cancer cells are unable to extract their own nutrient to feed themselves, finally starving to death. Cellular experiments, such as the measurements of extracellular lactate concentration and intracellular ATP level of cancer cells indicated that 2DG is efficient to block glucose metabolism and lead to starvation. In addition, immunoblotting of the autophagy indicator proteins, such as microtubule‐associated protein 1 light chain 3B (LC3B) and sequestosome 1 (p62), has evidenced significantly suppressed autophagic activity of cancer cells after BP treatment. In vivo results also demonstrate the synergetic effect between 2DG and BP nanosheets, resulting in remarkable super additive (“1 + 1 > 2”) therapeutic efficacy. Our future efforts aim to construct an integrated nanosystem coloaded with an antiglycolytic agent and an autophagy inhibitor to achieve optimal therapeutic effect by regulating the proportion between the two agents.

**Figure 11:**
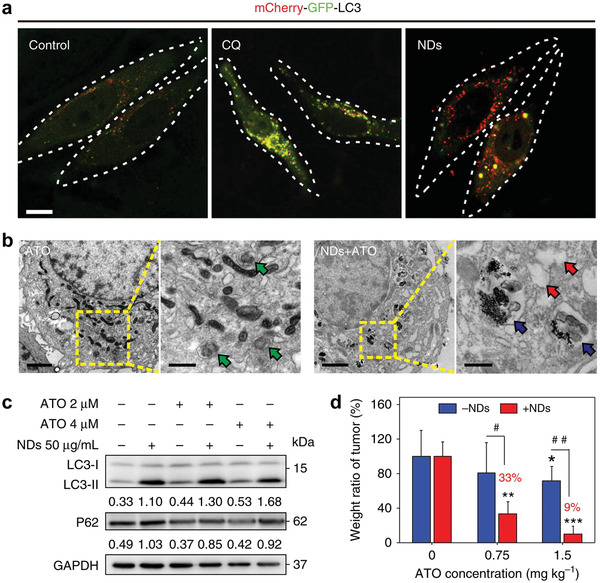
Nanodiamond as an autophagy inhibitor for allosterically enhancing the chemotherapeutic effect of arsenic trioxide. a) Confocal fluorescence images of cancer cells transfected with mCherry‐GFP‐LC3 after incubation with chloroquine (CQ) or nanodiamonds (NDs) for 48 h (yellow, autophagosomes; red, autolysosomes). Scale bar, 10 µm. b) Bio‐TEM images of cancer cells treated with single arsenic trioxide (ATO) or the mixture of NDs and ATO. Scale bars, 1 µm for left low magnification images, and 500 nm for right high magnification images. Green arrows indicate structures of autophagosomes induced by ATO, while blue arrows indicate autolysosome containing NDs induced by NDs/ATO mixture. Additionally, red arrows point to vacuoles inside cells. c) Immunoblot analyses revealing the expressions of autophagy‐related proteins LC3 and p62 in cancer cells. Glyceraldehyde 3‐phosphate dehydrogenase (GAPDH) was used as a loading control. d) Weight ratio of tumors harvested from mice after different treatments. **P < 0.01, ***P < 0.001, significantly different from normal saline; #*P* < 0.05, ##*P* < 0.01, significantly different from ATO. Reproduced with permission.^[^ ^42^ ^]^ Copyright 2018, Springer Nature.

Cancer cells can detoxicate themselves to resist extrinsic chemical perturbations by activating autophagy to degrade toxic components and re‐establish homeostasis.^[^ ^41^ ^]^ Therefore, in addition to previous tumor‐starving therapy, other therapeutic modalities such as chemotherapy, nanocatalytic therapy, PDT, etc., may also be largely compromised by autophagy. Using nanomaterials or pharmaceuticals to block autophagic flux is expected to sensitize cancer cells to therapeutic interventions and resultantly elevate therapeutic efficacies. For example, arsenic trioxide is an emerging chemotherapeutic drug for blood cancers, but its efficacy in solid tumors is limited due to its autophagic degradation in various solid tumor cells. Fan et al. first demonstrated that nanodiamond could serve as a potent autophagic inhibitor to allosterically improve the chemotherapeutic efficacy of arsenic trioxide in solid tumors (**Figure** **11**).^[^ ^42^ ^]^ Mechanism responsible for the autophagy‐inhibiting capability of nanodiamond involves the blockage of nuclear protein 1, which is a transcriptional regulator for autolysosomal clearance regulation in cells. The combination of the two agents in liver tumor mice model resulted in 91% tumor volume reduction compared with that of 28% without nanodiamonds addition, further confirming the feasibility of this strategy. Very recently, Gao et al. also constructed a composite nanosystem coloaded with chemodrug DOX and the autophagy inhibitor hydrochloroquine (HCQ) for enhanced chemotherapy of glioma, and the therapeutic outcome can be further improved by using anti‐PD‐L1 antibody to modulate the immunosuppressed glioma microenvironment.^[^ ^43^ ^]^

**Figure 12:**
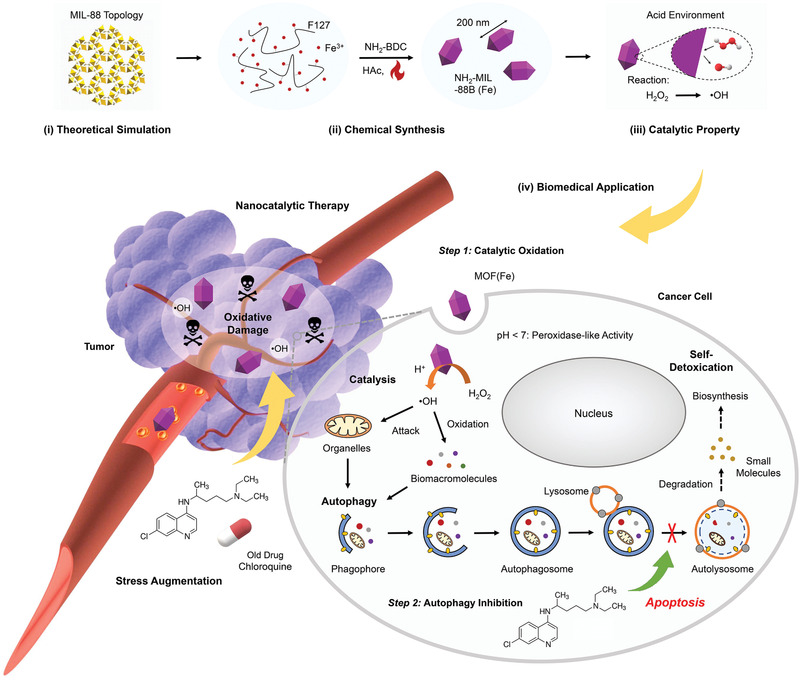
Pharmacological autophagy inhibition for augmenting the potency of MOF (Fe)‐based nanocatalytic cancer therapy. Schematic illustration for the material chemistry of MOF (Fe) and the combinational therapeutic concept. NH_2_‐BDC, 2‐aminoterephtalic acid; HAc, acetic acid. Reproduced with permission.^[^ ^46^ ^]^ Copyright 2020, Wiley‐VCH.

Nanocatalytic medicine has been developed recently for tumor‐specific therapy by initiating intratumoral catalytic reactions for in situ toxic species production.^[^ ^44^ ^]^ Various nanocatalysts, such as nanozymes with peroxidase‐like activities, have been introduced to trigger Fenton‐like reactions specifically in tumor regions, generating highly toxic •OH for tumor suppression.^[^ ^45^ ^]^ However, such a strategy could only lead to a limited therapeutic effect due to the protective effect of autophagy, by which the damaged cellular proteins and organelles after •OH attack are degraded in lysosomes for detoxification and nutrient cycling. To overcome this limitation, very recently we have designed a combined therapeutic strategy by concurrent uses of MOF (Fe) nanoparticles and chloroquine (CQ) in tumor (**Figure** **12**),^[^ ^46^ ^]^ of which MOF (Fe) nanoparticles with peroxidase‐like activity can convert intratumoral H_2_O_2_ into •OH via Fenton‐like reactions, triggering local oxidative stress, while CQ as a typical autophagy inhibitor can deacidify lysosomes to block the fusion of autophagosomes with lysosomes, preventing the degradation of toxic cellular components after oxidative damage. In this scenario, cancer cells can no longer detoxicate themselves to resist nanocatalytic therapy, finally leading to apoptotic death. In vivo experiments also manifested that the antitumor effect of synergistic therapy is much more distinct than the linear addition of those of single MOF (Fe) or CQ (78% vs 50% for A375 tumor xenografts, 74% vs 54% for HeLa tumor xenografts), evidencing the feasibility of this combined strategy. This work provides a general approach to enhance nanocatalytic therapy, which may further promote the development of this emerging area.

**Figure 13:**
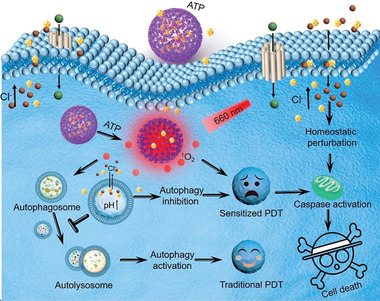
Autophagy inhibition by an ATP‐responsive nanosystem for sensitizing cancer cells to PDT. The squaramide loaded in the nanoparticles enables coupling transport of H^+^/Cl^−^ across the lysosomal membrane, resulting in lysosome alkalization and autophagy inhibition. Reproduced with permission.^[^ ^47^ ^]^ Copyright 2019, American Chemical Society.

The significant reduction of the chemoresistance of cancer cells after autophagy inhibition indicates that other ROS‐generating cancer therapeutic modalities, such as PDT, can also be enhanced by blocking autophagic flux. Zhang et al. have fabricated a porphyrinic porous coordination network (PCN) nanosystem incorporated with squaramide (SQU), which can respond to high levels of ATP in cancer cells and release SQU (**Figure** **13**).^[^ ^47^ ^]^ The SQU can mediate the transport of H^+^/Cl^−^ across the lysosomal membrane to deacidify lysosomes, resulting in suppressed autophagy. As a consequence, cancer cells are sensitized to the PDT triggered by the 660 nm NIR light, finally the tumor growth has been suppressed after this therapy. Yu et al. also used the autophagy inhibitor 3‐methyladenine to enhance zinc phthalocyanine nanoparticle‐induced PDT of osteosarcoma, and the synergy between the two agents also promote the down‐regulation of PD‐L1 favoring the enhancement of immunotherapy.^[^ ^48^ ^]^

The mechanism of autophagy is complex, but with the development of chemistry an increasing numbers of autophagy regulators have been developed in the past two years for harnessing such a catabolic process. It is expected that more autophagy‐regulating strategies will be developed for improving the efficacies of nanomedicines.

## 5 Additional Pathways

In addition to the aforementioned pathways, several other metabolic pathways, such as glutaminolysis, asparagine metabolism, lipolysis, and nicotinamide adenine dinucleotide (NAD) metabolism, also play important roles in cancer bioenergetics and biosynthesis.^[^ ^16^ ^]^ However, these pathways have been underestimated by chemists for a long time, which in fact could be developed as targets for cancer therapy. For example, glutamine is the most abundant amino acid in plasma and a key component of mammalian tissue matrix. It serves a substrate for protein synthesis, and its amine groups can also be used to generate other nonessential amino acids through transamination. Moreover, glutamine can replenish TCA cycle intermediates through anapleurosis, enabling its participation in anabolic processes.^[^ ^16^ ^]^ Cancer cells rely heavily on glutaminolysis to sustain their biosynthesis for tumor growth, during which large amounts of glutamine are converted into glutamate then *α*‐ketoglutarate.^[^ ^49^ ^]^ Due to the importance of glutaminolysis in tumor metabolism, the inhibition of glutaminolytic pathway is expected to be effective in tumor regression.

**Figure 14:**
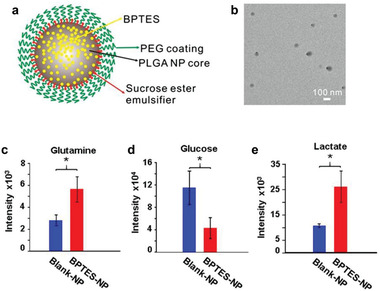
Combination of glutaminolysis and glucose metabolism inhibitions for pancreatic cancer treatment. a) Component of the designed nanoparticle encapsulating the glutaminolysis inhibitor bis‐2‐(5‐phenylacetamido‐1,2,4‐thiadiazol‐2‐yl)ethyl sulfide (BPTES). b) TEM image of the BPTES nanoparticles. c–e) Metabolomics analysis of c) glutamine, d) glucose, and e) lactate of tumors harvested from mice treated with BPTES nanoparticles. Reproduced with permission.^[^ ^50^ ^]^ Copyright 2016, National Academy of Sciences.

Elgogary et al. first encapsulated bis‐2‐(5‐phenylacetamido‐1,2,4‐thiadiazol‐2‐yl)ethyl sulfide (BPTES), an insoluble glutaminolysis inhibitor, in poly (lactic‐*co*‐glycolic acid) (PLGA) nanoparticles for the treatment of pancreatic cancer (**Figure** **14**).^[^ ^50^ ^]^ BPTES can inhibit the activity of glutaminase, an enzyme catalyzing the first step of glutaminolysis (i.e., the conversion of glutamine to glutamate), by binding at the oligomerization interface of the glutaminase tetramer, resulting in a decreased glutamine consumption. However, the treatment of BPTES‐loaded nanoparticle only led to a mild antitumor effect due to the biological fact that cancer cells can adapt to the metabolic alterations by switching between glycometabolism and glutaminolysis, leading to an increased activity of glycometabolism to compensate for the energy and nutrient deficiencies triggered by glutaminolysis inhibition. Therefore, in this works the nanoparticles were further used in conjunction with metformin, the most prescribed antihyperglycaemic agent that can inhibit the mitochondrial respiration by interfering with mitochondrial complex I.^[^ ^5^ ^]^ The combination therapy resulted in much more significant pancreatic tumor regression than either single treatment alone, revealing that the glycometabolism and glutaminolysis of cancer cells should be inhibited concurrently for better therapeutic outcome when we design future antimetabolic therapy against tumor. Inspired by this work, very recently, Li et al. have also achieved codelivery of 2DG and a glutaminolysis inhibitor V9302 via a micellar formulation for synergistic cancer therapy.^[^ ^51^ ^]^

The success of glutamine‐targeting therapy has aroused the enthusiasm of scientists to explore nanotherapies targeting other amino acids. For example, although asparagine is a nonessential amino acid that can be readily synthesized by human bodies, however, several types of cancer such as acute lymphoblastic leukaemia (ALL) are functional asparagine auxotrophs.^[^ ^6^ ^]^ Therefore, developing asparagine‐depleting approach is expected to promote tumor suppression. L‐asparaginase is an enzyme that can deaminate asparagine to aspartic acid, which has been developed to treat ALL by limiting the asparagine availability of cancer cells. Mou et al. also loaded asparaginase plasmid in MSNs to favor the expression of L‐asparaginase in cancer cells, leading to significant cytotoxicity.^[^ ^52^ ^]^ Further in vivo experiments are required to evaluate the feasibility of this nanotherapy in a more comprehensive way.

**Figure 15:**
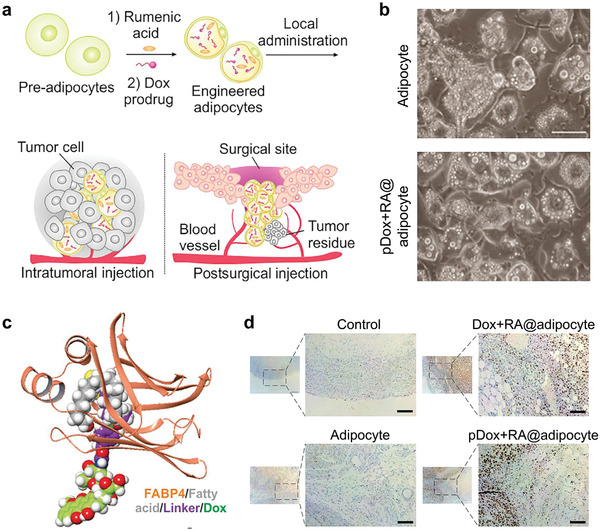
Leveraging tumor lipid metabolism by engineered adipocytes for cancer treatment. a) Schematic illustration for the adipocyte‐engineering and subsequent therapeutic process. b) Images of normally differentiated adipocytes (up) and engineered adipocytes (down). Scale bar, 200 µm. pDox, a doxorubicin prodrug; RA, rumenic acid, an anticancer fatty acid. c) Simulation of pDox and the fatty acid‐binding protein 4 (FABP4) binding. d) TUNEL staining of tumors after different treatments. Reproduced with permission.^[^ ^54^ ^]^ Copyright 2019, Elsevier.

Beyond the scope of glycometabolism and amino acid metabolism, the lipid metabolism of cancer cell has also attracted the attention of scientists to develop new cancer therapeutic modalities. Fatty acids are a prominent source of anabolic substrates as well as reducing equivalents.^[^ ^5^ ^]^ Cancer cells can utilize extrinsic fatty acids from the tumor microenvironment and tumor‐associated adipocytes through the fatty acid‐binding protein 4 (FABP4) and tumor cell‐induced lipolysis.^[^ ^53^ ^]^ Different from the aforementioned therapeutic strategies that aim to inhibit metabolic pathways of cancer cells, Gu et al. recently developed engineered adipocytes to exploit lipid metabolism for cancer therapy (**Figure** **15**).^[^ ^54^ ^]^ In this study, rumenic acid (RA), an anticancer fatty acid, and a DOX prodrug were encapsulated in adipocytes to deliver these therapeutics to tumor. After intratumoral or postsurgical administration, the lipolysis of cancer cells promotes the release of RA and pDox from the engineered adipocytes, exerting antitumor effect while downregulating programmed cell death‐ligand 1 (PD‐L1) expression, favoring CD4^+^ and CD8^+^ T lymphocyte‐mediated immunotherapy. This strategy by leveraging tumor lipolysis may benefit the development of lipolysis‐based cancer therapeutics.

Ferroptosis is a new form of cell death resulting from aberrant lipid metabolism.^[^ ^55^ ^]^ Recent efforts in nanotechnology have also been devoted to the design of nanoplatforms for promoting tumor ferroptosis.^[^ ^56^ ^]^ As glutathione peroxidase 4 (GPX4) is an important regulator of cancer cell ferroptosis,^[^ ^57^ ^]^ Gao et al. have prepared a polymer micelle system loaded with RSL3, a GPX4 inhibitor, for triggering ferroptosis and reverse multidrug resistance.^[^ ^58^ ^]^ More significant works are expected in the future to harness lipid metabolism and tumor ferroptosis effectively for further elevating the cancer‐therapeutic outcome.

**Figure 16:**
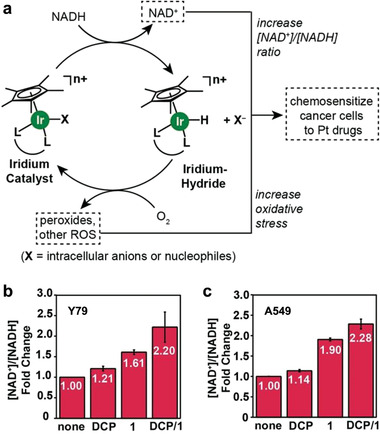
Organoiridium catalysts regulate celluar NAD^+^/NADH ratio for sensitizing cancer cells to chemodrugs. a) Organoiridium catalyst‐triggered chemical reactions lead to increased chemosensitivity of cancer cells toward platinum drugs. b,c) [NAD^+^]/[NADH] ratio change in Y79 (eye/retina cancer) and A549 (lung cancer) cells upon treatment with carboplatin (denoted DCP), complex 1 (denoted 1), or both. Reproduced with permission.^[^ ^60^ ^]^ Copyright 2017, Wiley‐VCH.

NAD is an essential cofactor and a primary electron carrier in various metabolic oxidation‐reduction reactions of cells, such as glycolytic reactions and OXPHOS.^[^ ^59^ ^]^ It has oxidized (NAD^+^) and reduced (NADH) forms, and the alterations in [NAD^+^]/[NADH] ratio may affect various metabolic processes and lead to decreased cell viability. The cancer therapeutic potential of NAD metabolism regulation was first explored by Yang et al., who prepared an organoiridium catalyst that could convert NADH into NAD^+^ via catalytic transfer hydrogenation (**Figure** **16**).^[^ ^60^ ^]^ Such an increase in [NAD^+^]/[NADH] ratio led to enhanced sensitivity of cancer cells toward platinum drugs such as carboplatin, while the viability of normal cells was unaffected after the combined treatments of organoiridium catalyst and carboplatin. As NADH is required in both glycolysis and OXPHOS respectively catalyzed by LDH and mitochondrial complex I,^[^ ^6^ ^]^ such a therapeutic approach is believed to be capable of inhibiting glycometabolism and thus overcoming chemoresistance of cancer cells. More evidences are also required to reveal the mechanism of the specificity of this therapeutic strategy against cancer.

## 6 Conclusions

Tumor metabolism has aroused broad attentions among researchers during the past several years. The alterations in metabolic levels confers cancer cells with strengthened resistance against various therapeutic interventions, such as chemodrugs, oxidative stress, hyperthermia, etc. Therefore, when we design nanotherapeutics, we should pay enough attentions to such a metabolic characteristic of cancer cells to achieve expected therapeutic efficacy. In this review, we have focused on the characteristic metabolic pathways of cancer cells, such as aerobic respiration, glycolysis, autophagy, glutaminolysis, asparagine metabolism, lipolysis, and NAD metabolism, to provide a comprehensive summery on the very recent advances in the design of nanomedicines that can regulate tumor metabolism for substantially enhancing conventional therapeutic modalities, such as photodynamic therapy, photothermal therapy, nanocatalytic therapy, tumor‐starving/suffocating therapy, etc. The mechanism by which these nanomedicines regulate tumor metabolism has been systematically discussed in this review, and the synergy between metabolism regulation and therapeutic interventions has also been elucidated in detail.

This field grows fast, and is regarded as a new subdiscipline of nanomedicine. However, several issues should be distinctively addressed, for benefiting the further development of this emerging area:

With the development of nanotechnology, various nanocarriers, such as liposomes, MSNs and MOF nanoparticles, have been applied to improve the bioavailability of drug molecules. Recent advances in the metabolism regulations and antimetabolites have provided us with a large warehouse to select proper antimetabolic drugs to be combined with multifunctional nanosystems for achieving improved therapeutic outcome. Importantly, a large number of current antimetabolites have been approved by the US Food and Drug Administration (FDA) or entered clinical trials, such as metformin, 2DG, CQ, L‐asparaginase, etc., with predictable biological behaviors and reliable biosafety profiles,^[^ ^6^ ^]^ it is expected that nanomedicines engineered with these antimetabolites can also be processed for clinical translation in the future, based on a comprehensive knowledge of the chemical and biological properties of these composite nanoplatforms in in vivo models.

1) The metabolic pathways of cancer cells are complex but present us abundant targets, such as metabolites and enzymes, for metabolism regulation. Till now, we have only explored several targets in several metabolic pathways while numbers of other metabolic targets have not been exploited for augmenting nanomedicines. For example, pentose phosphate pathway (PPP, also known as phosphogluconate pathway or hexose monophosphate shunt) is a metabolic circuitry that converts glycolytic intermediates into pentoses (5‐carbon sugars) and nicotinamide adenine dinucleotide phosphate (NADPH), which is important for nucleotides synthesis of neoplastic cells.^[^ ^5^ ^]^ Nanomedicines targeting the metabolic flux through the PPP may also be promising for triggering potent antitumor effect. In addition, for the main metabolic pathways of cancer cells that have been explored as the targets of nanomedicines, such as glycolysis, OXPHOS, autophagy, etc., there are still many metabolites and enzymes being unexplored. Future researches may concentrate on these metabolic pathways to develop effective therapeutic approaches.
2) A better understanding on cancer metabolism is still necessary for us to clarify the interdependent relationship between different metabolic pathways and to design precision chemical strategies for tumor suppression. As these pathways intertwine with each other the regulation of one single pathway by nanomedicines may affect the metabolic flux of other pathways, while cancer cells may correspondingly adapt their metabolic pathways to alternative ones to compensate for the energy and nutrient deficiency triggered by the blockage of the initial pathway. As discussed in the previous section, cancer cells can switch their metabolic pathways between glycolysis and OXPHOS, as well as between glycometabolism and glutaminolysis. Therefore, when we design metabolism‐regulating nanomedicines, we are supposed to concurrently inhibit several related pathways in one treatment for improved therapeutic effects. This requires the well‐tailored multifunctional nanomedicines to achieve cooperative metabolism‐regulating effects on different pathways.
3) The chemistries of nanomaterials have been extensively investigated in the past decade, and several of nanomaterials have been demonstrated with intrinsic metabolism‐regulating capabilities, such as Au nanoparticles (glucose deprivation), Ag nanoparticles (OXPHOS inhibition), BP nanosheets (autophagy inhibition), nanodiamonds (autophagy inhibition), etc. It is expected that more inorganic nanoparticles will be discovered with metabolism‐regulating capabilities in the future, offering more options in designing cancer nanotherapeutics. Owing to the intrinsic multifunctionality of inorganic nanoparticles, such as photosensitiveness for favoring photodynamic ROS generation, photothermal conversion capability for enabling PTT, X‐ray attenuation ability for enabling radiation therapy and CT imaging, this category of nanomedicines are promising candidates in the future metabolism regulation‐based synergistic therapies as well as theranostics.
4) The pharmacokinetics and other biosafety profiles of these metabolism‐regulating nanomedicines should also be under extensive investigations to guarantee low systemic side effect. As some types of highly proliferating normal cells metabolize in a similar fashion to cancer cells,^[^ ^5^ ^]^ the administrations of these nanomedicines will inevitably result in adverse effects on the metabolism of these normal cells. Therefore, great efforts should be devoted in the future to further improve the cancer‐specificity of these metabolism‐regulating nanomedicines, thus mitigating their side effect on normal tissues and organs. We should position the therapeutic effect on tumor by taking full advantage of the nanomaterial chemistry, as well as the cell biology, while current diagnostic modalities may assist us to achieve more precision spatiotemporal control of therapeutic interventions for improved outcome. The biosafety issue of thee nanomedicines is also important for their future clinical translation.

As the aforementioned key scientific issues in the development of metabolism‐regulating nanomedicines are becoming better addressed, it is expected that tumor therapeutic outcomes will be significantly improved, benefiting the well‐being of humans.

## Conflict of Interest

The authors declare no conflict of interest.
